# Efficient Production of Scleroglucan by *Sclerotium rolfsii* and Insights Into Molecular Weight Modification by High-Pressure Homogenization

**DOI:** 10.3389/fbioe.2021.748213

**Published:** 2021-09-03

**Authors:** Weizhu Zeng, Junyi Wang, Xiaoyu Shan, Shiqin Yu, Jingwen Zhou

**Affiliations:** ^1^ National Engineering Laboratory for Cereal Fermentation Technology, Jiangnan University, Wuxi, China; ^2^ Science Center for Future Foods, Jiangnan University, Wuxi, China; ^3^ Key Laboratory of Industrial Biotechnology, Ministry of Education, School of Biotechnology, Jiangnan University, Wuxi, China; ^4^ Jiangsu Provisional Research Center for Bioactive Product Processing Technology, Jiangnan University, Wuxi, China

**Keywords:** *Sclerotium rolfsii*, scleroglucan, fed-batch, molecular weight, high-pressure homogenization

## Abstract

Scleroglucan is a non-ionic water-soluble polysaccharide, and has been widely used in the petroleum, food, medicine and cosmetics industries. Currently, scleroglucan is mainly produced by *Sclerotium rolfsii*. A higher level of scleroglucan (42.0 g/L) was previously obtained with *S. rolfsii* WSH-G01. However, the production of scleroglucan was reduced despite a higher glucose concentration remaining. Additionally, the molecular weight of scleroglucan was large, thus restricted its application. In this study, by adjusting the state of seeds inoculated, the degradation issue of scleroglucan during the fermentation process was solved. By comparing different fed-batch strategies, 66.6 g/L of scleroglucan was harvested by a two-dose fed-batch mode, with 53.3% glucose conversion ratio. To modify the molecular weight of scleroglucan, a combination method with HCl and high-pressure homogenization treatment was established. Finally, scleroglucan with molecular weight of 4.61 × 10^5^ Da was obtained. The developed approaches provide references for the biosynthesis and molecular weight modification of polysaccharides.

## Introduction

Scleroglucan is a microbial exopolysaccharide, and is a typical β-glucan (Bai et al., 2021; Valdez et al., 2021). It consists of backbone β-1,3-linked-glucopyranosyl residues and branches of β-1,6-linked-glucopyranosyl residues. Due to its special structure of every three sugar residues of the main chain bearing a single β-1,6-linked-glucopyranosyl residue, the branching degree of scleroglucan is up to 0.33 (Castillo et al., 2015). This characteristic of high branching frequency endows scleroglucan with high water solubility different to other types of β-glucans. In addition, scleroglucan is reported to have a higher molecular weight (Tan et al., 2019; Elsehemy et al., 2020). Due to these outstanding properties of a unique chemical structure and higher molecular weight, scleroglucan has significant advantages in terms of water solubility, biocompatibility, pseudoplasticity, resistance to hydrolysis, salt tolerance, moisture retention and viscosity stability (Barcelos et al., 2020; Song et al., 2020). It has been applied in the petroleum, food, medicine and cosmetics industries (Giavasis, 2014; Li et al., 2020c).

Scleroglucan is mainly produced by the genus *Sclerotium* sp., including *S. rolfsii*, *S. glucanicum*, and *S. delphinii* (Schmid et al., 2011; Gao et al., 2021). *S. rolfsii* is the dominant producer, which can accumulate high concentrations of scleroglucan with diverse substrates, such as glucose, sucrose, xylose and molasses (Survase et al., 2007b; Taskin et al., 2010; Leonor Valdez et al., 2019). Strategies have been introduced to enhance its production, most of which were focused on the selection of medium components and cost-effective optimization of controlling fermentation conditions (Survase et al., 2007a; Zhang et al., 2017; Valdez et al., 2021). In our previous research, 42.0 g/L of scleroglucan was obtained with the screened strain *S. rolfsii* WSH-G01 (Tan et al., 2019). The molecular weight of the obtained scleroglucan reached 10^8^ Da and even 10^9^ Da, while application of the polysaccharide was greater when its molecular weight was below 10^6^. The function of scleroglucan was reported to be closely related to its molecular weight. Water solubility is poor when the molecular weight is too large, while the physiological function is lost when the molecular weight is too low (Kulicke et al., 1997; Farina et al., 2001; Castillo et al., 2015). Therefore, the modification of scleroglucan to obtain an ideal molecular weight is significant for improving its water solubility while maintaining its original function.

Currently, the commonly used methods for polysaccharide hydrolysis include physical hydrolysis, acid hydrolysis and enzymatic hydrolysis (He et al., 2018; Hu et al., 2021). Physical hydrolysis is a relatively fast and clean method, and includes microwave, irradiation and ultrasonication; however, the application of these methods is limited due to low yield and high cost (Liu et al., 2021a). Recently, high-pressure homogenization (HPH) as an emerging technology was used for polysaccharide hydrolysis (Belmiro et al., 2018; Xie et al., 2021). Acid hydrolysis is usually used for the degradation of polysaccharides by controlling the pH value. However, this method has some drawbacks, such as a wide distribution of product molecular weight and poor homogeneity formation (Lin et al., 2017). Enzymatic hydrolysis is considered the optimal choice for the high regional-selectivity and stereoselectivity of enzymes, but it also requires special conditions for storage, reaction and removal from the polysaccharide system (Cristina Vallejo-Garcia et al., 2019; Zheng et al., 2020). For modification of the molecular weight of scleroglucan, a suitable hydrolase with efficient capacity for scleroglucan degradation was not found after the expression and identification of β-glucanases from different microorganisms (Zeng et al., 2021). Co-culture with *Pichia pastoris* GS115 expressed an endo-β-1,3-glucanase (glycoside hydrolase family 55) from *Trichoderma harzianum* with *S. rolfsii* WSH-G01, and the final polymerization degree was only 2–17 (Gao et al., 2021), which did not fulfill the common requirement that the molecular weight be between 10^5^ and 10^6^ Da.

In *S. rolfsii*, it was reported that β-glucanases and β-1,3-glucanases were expressed to degrade scleroglucan in the late fermentation stage (Farina et al., 2009; Tan et al., 2019). Based on the whole genome sequencing, some β-glucanases and β-1,3-glucanases were discovered and then overexpressed in *Pichia pastoris* in our previous study. However, the results showed that the hydrolysis effects of these identified β-glucanases on scleroglucan degradation were extremely weak (Zeng et al., 2021). In addition, the utilization of the acid hydrolysis method alone to obtain the appropriate molecular weight of modified scleroglucan was also ineffective. In the present study, the fermentation process was optimized to further enhance scleroglucan production. Furthermore, the scleroglucan degradation method based on HPH treatment was investigated to obtain the appropriate molecular weight of scleroglucan. Finally, 66.6 g/L of scleroglucan was produced by a two-stage fed-batch fermentation strategy. In addition, an HCl-HPH combination method was also established for scleroglucan degradation, which resulted in the molecular weight of scleroglucan being degraded to 4.61 × 10^5^ Da. The methods developed in this study could provide a reference for the efficient fermentation, production and degradation of scleroglucan and other polysaccharides.

## Materials and Methods

### Strains

The wild-type strain *S. rolfsii* WSH-G01 (CCTCC M2017646), which is a scleroglucan overproducer screened in our previous work, was used in this study (Gao et al., 2018).

### Medium and Culture Conditions

The medium for slant, seed cultures contained the following: 30.0 g/L glucose, 1.0 g/L yeast extract, 3.0 g/L NaNO_3_, 1.0 g/L KH_2_PO_4_, 0.5 g/L KCl, and 0.5 g/L MgSO_4_·7H_2_O, at pH 4.0. In the slant medium, 20 g/L agar was added. The fermentation medium contained the following: 95.0 g/L initial glucose, 1.0 g/L yeast extract, 3.0 g/L NaNO_3_, 0.5 g/L KCl, 0.5 g/L MgSO_4_·7H_2_O, 1.0 g/L KH_2_PO_4_, 1.5 g/L citric acid, at an initial pH of 4.0. All the components were autoclaved for 20 min at 115°C (Tan et al., 2019). In addition, different concentrations of glucose sterilized before addition to the medium were added based on the different fermentation controlling strategies.

*S. rolfsii* was activated on PDA (potato dextrose agar) plates at 30°C for 96 h and then inoculated into 500 ml shaking flasks containing 50 ml culture medium for 60 h at 200 r/min and 30°C on a reciprocal shaker (Zhichu, Shanghai, China). The fed-batch fermentation was performed in a 5 L fermenter (T&J Bioengineering, Shanghai, China) with a 3.5 L working volume at 400 r/min and 1.0 vvm (volume air per volume). The pH was controlled automatically by adding 4.0 mol/L NaOH or 4.0 mol/L HCl according to the different strategies. The size of the inoculation was 5% (v/v) and all cultivations were carried out at 30°C. Different fed-batch strategies are investigated. The initial glucose content was 95 g/L and the total concentration of glucose was 125 g/L. For the two-dose fed-batch mode, glucose was intermittently fed twice with 15 g/L each time at 36 and 60 h, respectively. For the three-dose fed-batch mode, glucose was intermittently fed three times with 10 g/L each time at 36, 48, and 60 h, respectively. For the constant rate feeding fed-batch mode, glucose was constantly fed at a rate of 1.25 g/(L·h) during 36–60 h. All fermentations were performed in triplicate and the results presented as mean values.

### Combined HCl-HPH Treatment of Scleroglucan

The pure sample of scleroglucan extracted from fermentation broth (the extraction method of scleroglucan was showed in *Analytical Method*) was re-dissolved in distilled water. Then, 0.20 mol/L of HCl was added and the solution was hydrolyzed in a water bath at 90°C for different times (2, 4, 6, 8, and 10 h). After cooling to room temperature, the pH of the hydrolysis system was adjusted to 7.0 with 2.0 mol/L NaOH. The HPH treatment was then conducted at 80 MPa for 25 s and the operation was repeated nine cycles.

### Analytical Method

Determination of dry cell weight (DCW): The samples of fermentation broth were diluted five times with distilled water and the pH was adjusted to 7.0 with 2.0 mol/L NaOH or 2.0 mol/L HCl. The diluent solutions were then centrifuged at 10,000 × *g* for 30 min after being heated at 80°C for 30 min in a water bath. The cells were washed three times with ultrapure water and then dried at 105°C to a constant weight.

Extraction of scleroglucan and determination of its production: The fermentation broths obtained at different time points were diluted 3-fold by distilled water and mixed well. The supernatant was then obtained by centrifuging at 10,000 × *g* after being heated at 80 C in a water bath for 30 min. The pH of the supernatant was adjusted to 7.0 with 2.0 mol/L NaOH or 2.0 mol/L HCl. After adding an equal volume of anhydrous ethanol, scleroglucan was harvested by the alcohol deposition method at 4 C for 16 h. The production of scleroglucan was determined after freeze-drying to constant weight.

Determination of the concentrations of glucose and organic acids: Glucose and oxalic acid were determined by high performance liquid chromatography (HPLC, Agilent 1260, CA, United States) with an Aminex HPX-87H column (Bio-Rad, CA, United States). The elution conditions were as follows: 5 mmol/L H_2_SO_4_ of mobile phase, 0.4 ml/min flow rate, 40°C column temperature and 10 µl injection volume. Glucose was detected by applying the differential refraction index detector, and oxalic acid was detected by an UV detector at 210 nm (Gao et al., 2018; Tan et al., 2019).

Determination of the molecular weight of the polysaccharide: The molecular weight of scleroglucan treated with HPH was determined by high performance gel penetration chromatography (HPGPC, Waters, MA, United States) with a Shodex OHpak SB-806M HQ (Shodex, Tokyo, Japan). The detection conditions were as follows: parallax detector, 0.1 mol/L NaNO_3_ of mobile phase, 1.0 ml/min flow rate, 40°C column temperature and 50 µl injection volume. The differential refraction index detector was used.

## Results and Discussion

### Solution of the Scleroglucan Degradation During the Fermentation Process

In a previous study, 42.0 g/L of scleroglucan was produced by *S. rolfsii* WSH-G01, and further enhancement of scleroglucan production was difficult. The production of scleroglucan was gradually decreased and the content of substrate glucose was increased in the late stage of the fermentation process (Tan et al., 2019). The expression of β-glucanases and β-1,3-glucanases was initially considered to be the main reason for this phenomenon, as studies have reported that *S. rolfsii* can express some glucanohydrolases that degrade scleroglucan into glucose, providing energy to maintain basic cell growth and metabolism (Bateman, 1972; Martin et al., 2007). However, the effect of β-glucanases on scleroglucan degradation was determined to be weak through mining the autologous β-glucanase genes in *S. rolfsii* (Zeng et al., 2021). In addition, it was reported that other factors could also affect accumulation of the target product during the fermentation process, such as the seed culture and fermentation conditions (Sun et al., 2020b; Tian et al., 2020).

**FIGURE 1:**
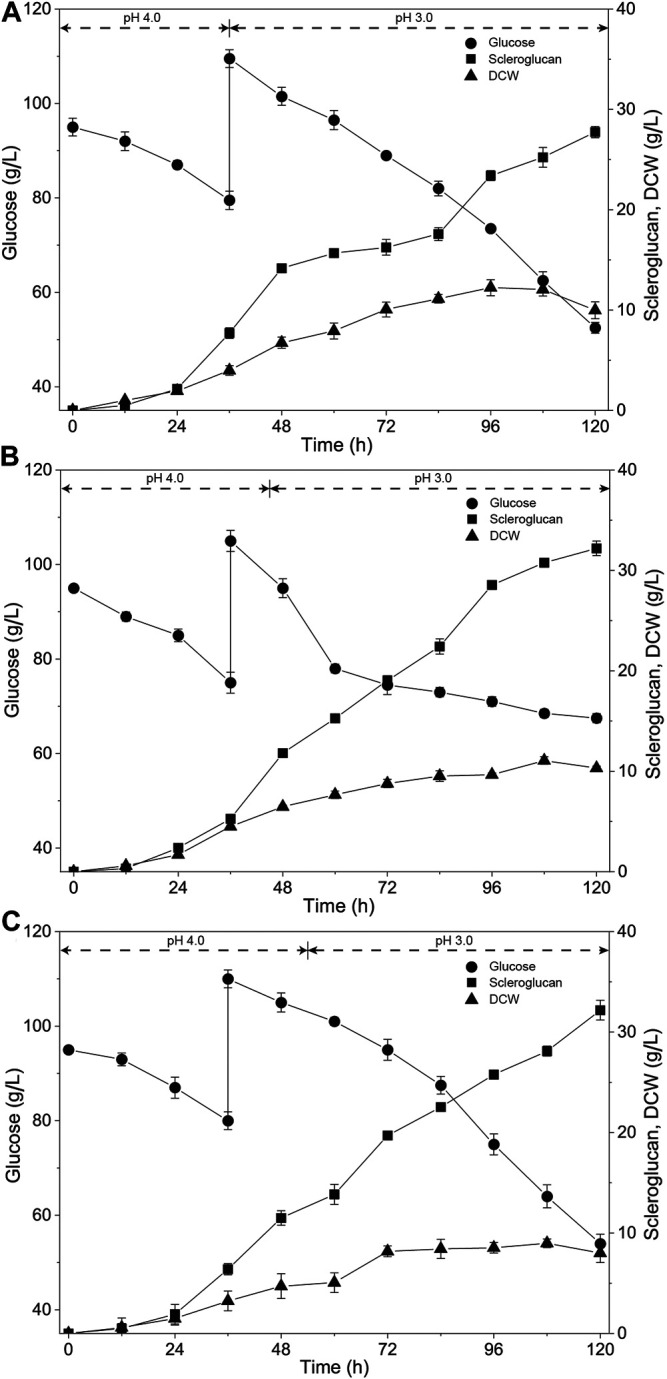
Time course of scleroglucan production using different pH-shift controlling fermentation strategies. The pH was controlled at 4.0 in the early fermentation process, and then adjusted to 3.0 at different times. **(A)** The pH was controlled at 4.0 before 36 h, and maintained at 3.0 after 36 h. **(B)** The pH was controlled at 4.0 before 45 h, and maintained at 3.0 after 45 h. **(C)** The pH was controlled at 4.0 before 54 h, and maintained at 3.0 after 54 h.

To solve the issue of scleroglucan degradation, adjustments to the fermentation process were introduced. The seeds inoculated were in the mid-log phase, and the seed culture time was adjusted from 72 to 60 h on the reciprocal shaker. In addition, to better suit the changed state of the seeds inoculated, three different time points for pH adjustment (36, 45, and 54 h) were compared based on a previously established pH-shift strategy and one-dose fed-batch strategy. The results are shown in Figure 1. The titer of scleroglucan was 27.7 g/L, 32.2 g/L and 32.2 g/L according to the three time points of pH adjustment at 36, 45, and 54 h, respectively. Compared with the previous results (Tan et al., 2019), the titer of scleroglucan was reduced, but the decrease in scleroglucan production did not appear in the late stage of the fermentation process. In addition, accumulation of the by-product oxalic acid was below 5 g/L. Considering the increased rate of scleroglucan in the late stage of the fermentation process, the time point for pH adjustment at 54 h was selected for the subsequent optimization process.

### Comparison of Different Fed-Batch Strategies on Scleroglucan Production

**FIGURE 2:**
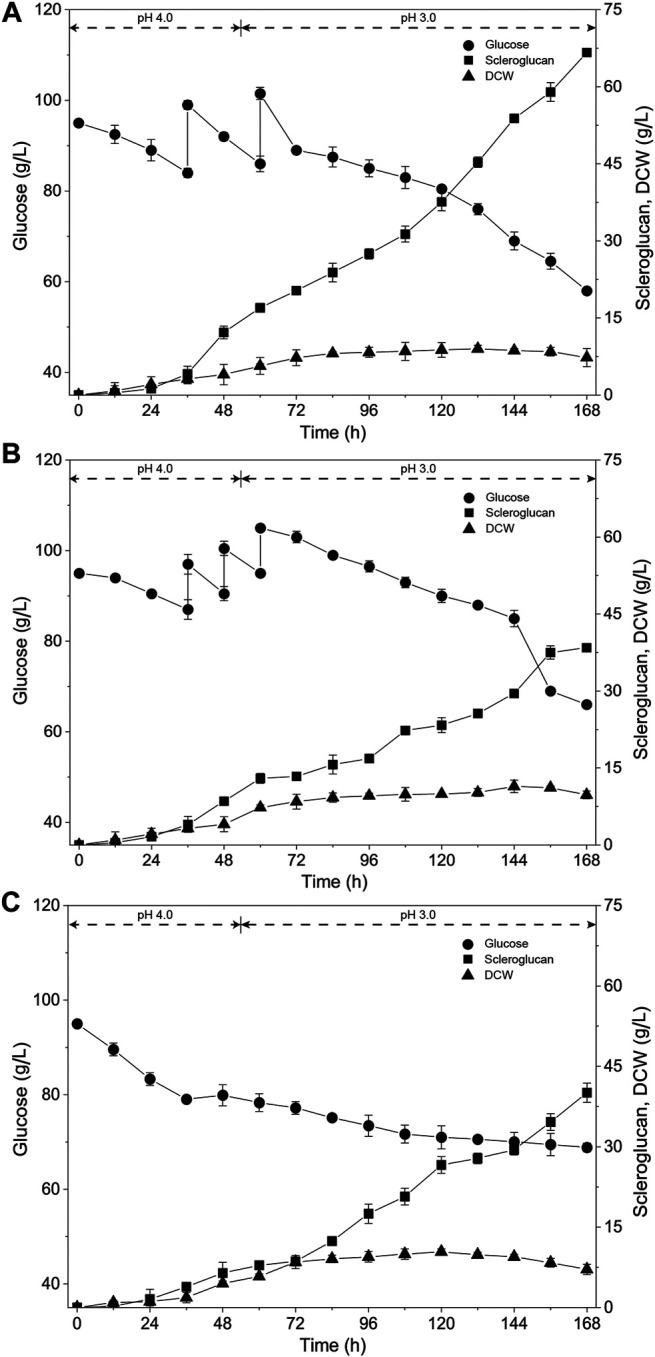
Time course of scleroglucan production using different feeding methods. The initial glucose concentration was 95.0 g/L and 30.0 g/L of glucose was added with different feeding approaches. **(A)** Two-dose fed-batch strategy (15.0 g/L of glucose was added at 36 and 60 h, respectively). **(B)** Three-dose fed-batch strategy, (10.0 g/L of glucose was added at 36, 48, and 60 h, respectively). **(C)** Constant speed feeding strategy (1.25 g/(L·h) of glucose was continuously added from 36 to 60 h).

To further enhance the production of scleroglucan, various feeding strategies were tested with the exception for the previously applied single-dose fed-batch mode. These strategies included the two-dose fed-batch mode, three-dose fed-batch mode and constant rate feeding fed-batch mode. The results are shown in Figure 2. The two-dose fed-batch mode yielded the best results, with the highest scleroglucan production being 66.6 g/L, a glucose conversion ratio of 53.3%, and productivity of 0.4 g/(L·h). Compared with the fed-batch process, the titer of scleroglucan was enhanced by 106.8%, and compared with the previous result (Tan et al., 2019), the titer of scleroglucan was enhanced by 58.7%.

The fed-batch mode is a usual strategy for improving the output of some target products, including the feeding of substrate or other ingredients. Different feeding modes also have diverse influences, such as the intermittent fed-batch mode and constant rate feeding fed-batch mode (Sun et al., 2020a; Liu et al., 2021b). In our previous research, diverse fed-batch strategies were established for specific products with different microorganisms, such as the one-dose fed-batch mode for enhancement of 2-phenylethanol (Tian et al., 2020), a multi-intermittent fed-batch mode for 2-keto-D-gluconic acid production (Zeng et al., 2019), and a constant rate feeding fed-batch mode for the simultaneous biosynthesis of α-ketoglutarate and pyruvate (Zeng et al., 2017). In this study, the production and glucose conversion efficiency were significantly enhanced with the intermittent fed-batch mode, while the productivity showed a slight decline. In the future, optimization could be conducted to further enhance the productivity and scleroglucan production, such as adding nitrogen sources, and improving the performance of strain and fermentation parameters.

### Effects of HPH Treatment on Scleroglucan Molecular Weight

Homogenization technology is the most widely used method for emulsifying and refining. The high-pressure homogenization (HPH) procedure possesses distinguishing advantages in high shear stress, high-frequency oscillation, and cavitation (Xie et al., 2018; Li et al., 2020b). It is often employed in food processing to reduce the particle size of solid substances and improve the quality of products, such as yogurt, fruit and vegetable juice (Wellala et al., 2020; Levy et al., 2021). HPH technology has been increasingly used in polysaccharide processing, and is mainly used to reduce the molecular weight of polysaccharides. For example, the structural characteristics of tamarind seed polysaccharides were obviously changed after HPH treatment, and therefore, affected the physicochemical properties of corn starch (Xie et al., 2021). In addition, it was reported that the viscosity of polysaccharides could also be reduced by HPH treatment (Villay et al., 2012).

**FIGURE 3:**
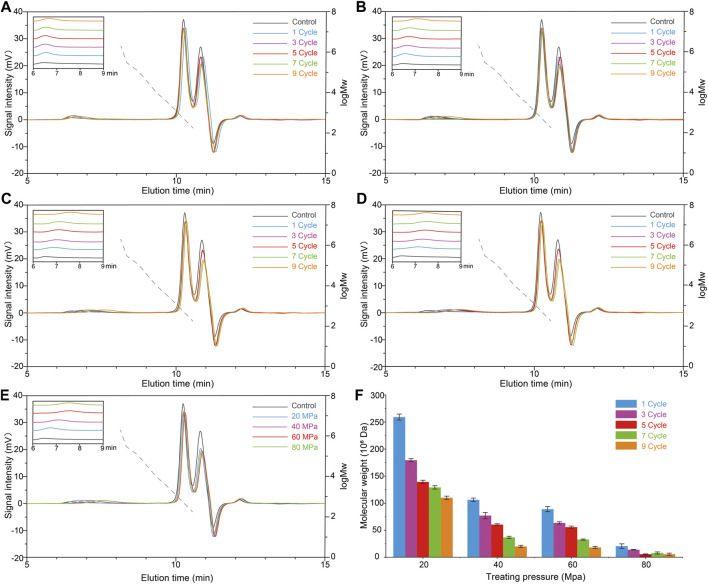
Effects of different pressures and cycles with HPH treatment on the molecular weight of scleroglucan. **(A)** Molecular weight (M_W_) chromatogram of scleroglucan treated with 20 MPa. **(B)** Molecular weight chromatogram of scleroglucan treated with 40 MPa. **(C)** Molecular weight chromatogram of scleroglucan treated with 60 MPa. **(D)** Molecular weight chromatogram of scleroglucan treated with 80 MPa. **(E)** Molecular weight chromatogram of scleroglucan treated with different pressures for nine cycles. **(F)** Distribution of scleroglucan in the molecular weight range of 10^6^–10^8^. The control was the sample not treated with HPH. The dotted lines represent the standard curve of scleroglucan molecular weight (1.80 × 10^2^–2.00 × 10^6^ Da). M_W_, Molecular weight.

To modify the molecular weight of scleroglucan for wider application value, the effects of HPH treatment on scleroglucan molecular weight was investigated. The content of scleroglucan in the sample was 1.0 g/L. The effects of different pressures (20, 40, 60, and 80 MPa) and different cycles (1, 3, 5, 7, and 9) were compared (Figures 3A–E). The result showed that HPH treatment has an effect on reducing the molecular weight of scleroglucan. The molecular weight of scleroglucan in the range of 10^6^–10^8^ was determined after HPH treatment, and the range of molecular weight was gradually degraded with increasing treatment pressure and cycle. A type of scleroglucan with a molecular weight of 5.6 × 10^6^ Da was obtained under the treatment conditions of 80 MPa and nine cycles. However, scleroglucan with the highest molecular weight was not degraded. Degradation was not obviously affected by increasing the number of cycles below 80 MPa (Figure 3F). In addition, it was not feasible to obtain the desired scleroglucan with lower molecular weight by further increasing the processing pressure, which was limited by the experimental equipment.

### Establishment of the HCl-HPH Combination Method for Scleroglucan Degradation

The molecular weight of the obtained scleroglucan was still too large, which was limited by utilization of the HPH treatment alone. Other strategies should be included to further modify scleroglucan. Recently, several combination methods with different single processes were established for polysaccharide modification, such as enzymatic hydrolysis combined with hydrothermal pretreatment for pectin (Wang et al., 2021), acid hydrolysis combined with an induced electric field for guar gum, chitosan and pectin (Li et al., 2020a). Acid hydrolysis with HCl, H_2_SO_4_ or organic acids is an effective method for polysaccharide degradation (Nguyen et al., 2020; Shi et al., 2020; Liu et al., 2021a). In this study, an HCl-HPH combination method was attempted to establish scleroglucan degradation. Based on the obtained conditions of HPH treatment, the parameters of HCl treatment were further optimized.

**FIGURE 4:**
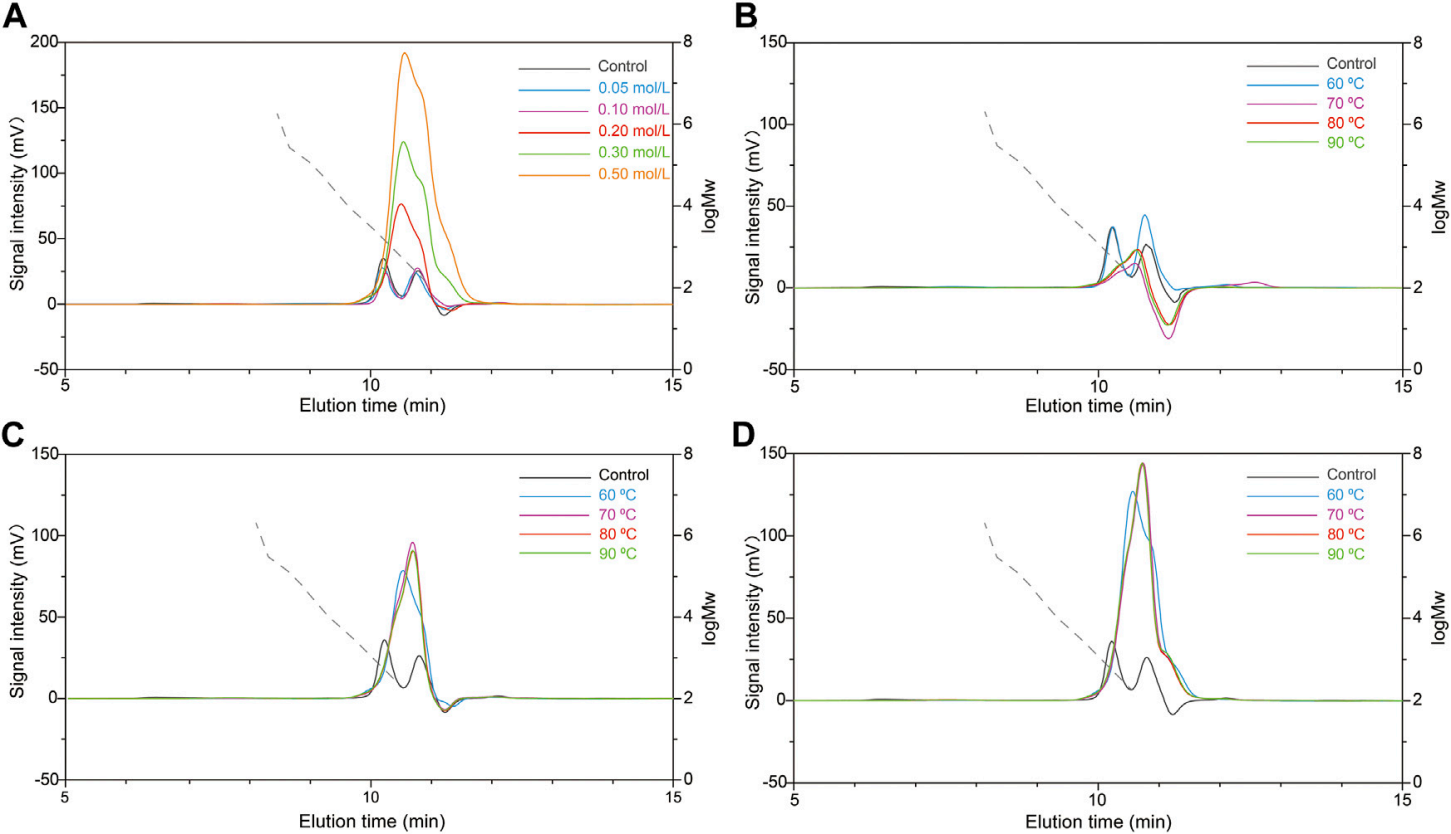
Effects of different HCl pretreatment conditions with the combination HCl-HPH method on molecular weight of scleroglucan. **(A)** Molecular weight chromatogram of scleroglucan pretreated with different concentrations of HCl (0.05, 0.10, 0.2, 0.30, and 0.50 mol/L) using the HCl-HPH method. **(B)** Molecular weight chromatogram of scleroglucan pretreated with 0.10 mol/L HCl using the HCl-HPH method at different temperatures. **(C)** Molecular weight chromatogram of scleroglucan pretreated with 0.20 mol/L HCl using the HCl-HPH method at different temperatures. **(D)** Molecular weight chromatogram of scleroglucan pretreated with 0.30 mol/L HCl using the HCl-HPH method at different temperatures. The control was the sample not pretreated with HCl. The dotted lines represent the curve of scleroglucan molecular weight (1.80 × 10^2^–2.00 × 10^6^ Da). M_W_, Molecular weight.

Firstly, the effects of different HCl concentrations (0.05, 0.1, 0.20, 0.30, and 0.50 mol/L) on the molecular weight of scleroglucan were investigated. Treatment with HCL only at 60°C for 2 h was not obtained the desired molecular weight of scleroglucan (Supplementary Table S1). Combining with HPH treatment, the molecular weight could be modified, and reduced to 1.62 × 10^6^ Da with 0.2 mol/L HCl pretreatment, but it still did not reach the desired range. It was found that the molecular weight decreased with increased HCl concentration at 0.05–0.20 mol/L, while it increased with continuous enhanced HCl concentration (Figure 4A, Supplementary Table S2). It was speculated that the higher molecular weight of scleroglucan was decomposed by high HCl concentrations, which usually has better capacity in polysaccharide degradation (Nguyen et al., 2020). Additionally, the degradation effects were further tested with different HCl concentrations (0.1, 0.2, and 0.3 mol/L) under higher treatment temperatures (70, 80, and 90°C) (Figures 4B–D). The results showed that the effect of pretreatment with 0.2 mol/L HCl at 90°C was best, producing scleroglucan of 1.32 × 10^6^ Da (Supplementary Table S3).

**FIGURE 5:**
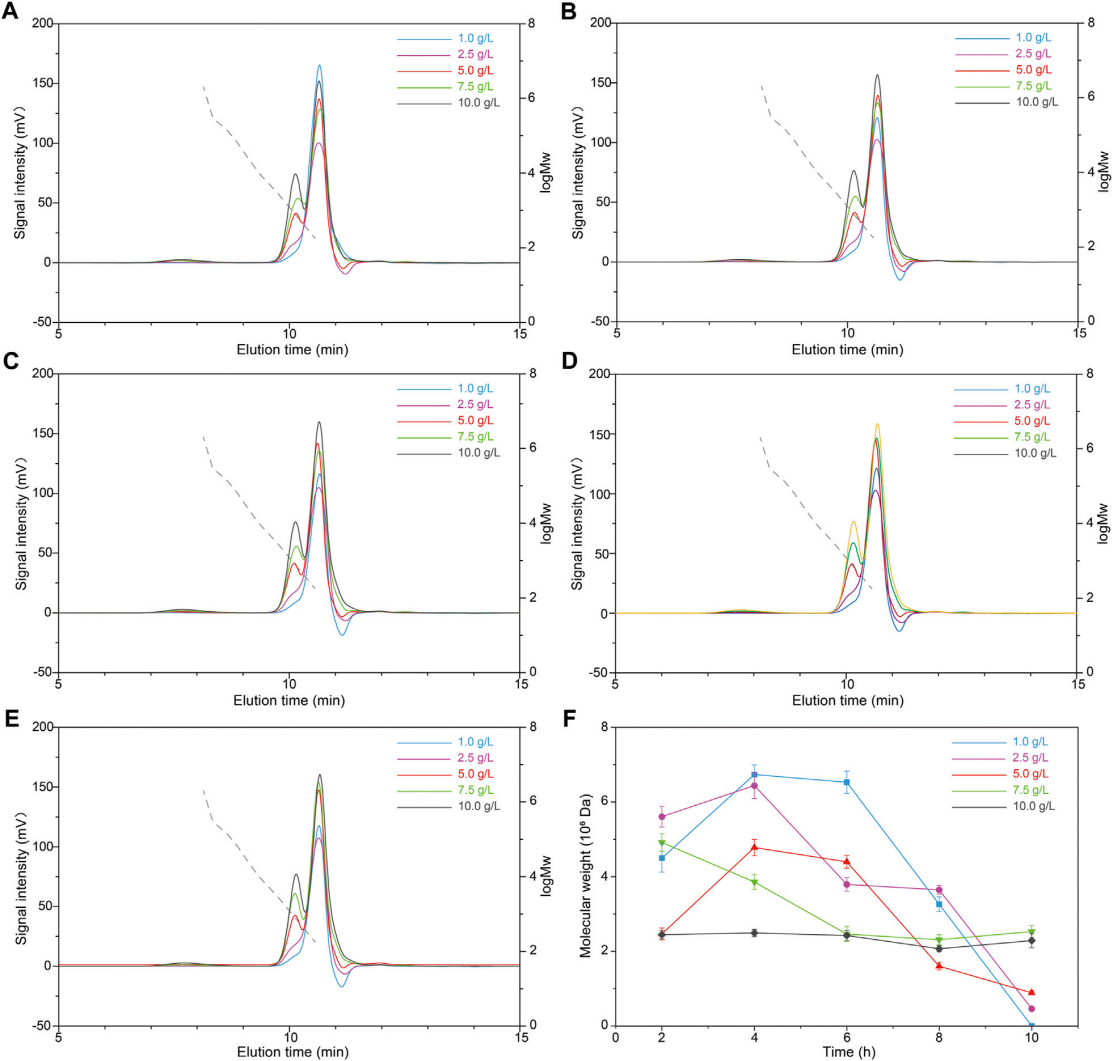
Effects of different scleroglucan concentrations with the combination HCl-HPH method on the molecular weight of scleroglucan. **(A)** Molecular weight chromatogram of different scleroglucan concentrations treated using the HCl-HPH method for 2 h. **(B)** Molecular weight chromatogram of different scleroglucan concentrations treated using the HCl-HPH method for 4 h. **(C)** Molecular weight chromatogram of different scleroglucan concentrations treated using the HCl-HPH method for 6 h. **(D)** Molecular weight chromatogram of different scleroglucan concentrations treated using the HCl-HPH method for 8 h. **(E)** Molecular weight chromatogram of different scleroglucan concentrations treated using the HCl-HPH method for 10 h. **(F)** Molecular weight change of scleroglucan (with high or medium molecular weight) treated using the HCl-HPH method for the samples with different scleroglucan concentrations. The dotted lines represent the curve of scleroglucan molecular weight (1.80 × 10^2^–2.00 × 10^6^ Da). M_W_, Molecular weight.

The influence of prolonged HCl pretreatment was further investigated. Simultaneously, different initial concentrations of scleroglucan (1.0, 2.5, 5.0, 7.5, and 10.0 g/L) with higher molecular weight were selected, which were extracted from samples in the middle of the scleroglucan fermentation process. Figure 5 shows the molecular weight changes in scleroglucan at different times with HCl pretreatment. The specific molecular weight of scleroglucan ranged from 10^5^ to 10^6^ Da and its peak proportion in the samples are shown in Table 1. These results showed that the established HCl-HPH combination method could be applied to modify scleroglucan. The desired molecular weight of modified scleroglucan was 4.61 × 10^5^ Da and 8.88 × 10^5^ Da by treating an initial 2.5 g/L of scleroglucan and 5.0 g/L of scleroglucan for 10 h, respectively, and the peak proportions were 4.80 and 2.55%, respectively. In the future, several optimizations should be introduced to enhance the peak proportion of the target modified scleroglucan. For example, because of limiting by the experimental equipment, the effect of higher processing pressure on the scleroglucan modification was not implemented in this study. The results showed that the degradation effect was obvious with the increased pressure, which could be considered for further dealing with the large amounts and high production. In addition, the enzymatic hydrolysis could also be combined with the established HCl-HPH method, though there are still no reports about enzymes that can efficiently degrade scleroglucan.

**TABLE 1:** Molecular weight of scleroglucan (10^5^–10^6^) and its peak proportion in the samples with different scleroglucan concentrations.

Conditions	Molecular weight (Da)	Peak proportion (%)
1.0 g/L scleroglucan treated for 2 h	4.50 × 10^6^	0.27
2.5 g/L scleroglucan treated for 2 h	5.61 × 10^6^	0.54
5.0 g/L scleroglucan treated for 2 h	2.46 × 10^6^	5.91
7.5 g/L scleroglucan treated for 2 h	4.92 × 10^6^	2.29
10.0 g/L scleroglucan treated for 2 h	2.45 × 10^6^	2.75
1.0 g/L scleroglucan treated for 4 h	6.74 × 10^6^	0.16
2.5 g/L scleroglucan treated for 4 h	6.44 × 10^6^	0.66
5.0 g/L scleroglucan treated for 4 h	4.78 × 10^6^	5.19
7.5 g/L scleroglucan treated for 4 h	3.86 × 10^6^	2.19
10.0 g/L scleroglucan treated for 4 h	2.49 × 10^6^	2.19
1.0 g/L scleroglucan treated for 6 h	6.53 × 10^6^	0.22
2.5 g/L scleroglucan treated for 6 h	3.79 × 10^6^	0.74
5.0 g/L scleroglucan treated for 6 h	4.40 × 10^6^	4.72
7.5 g/L scleroglucan treated for 6 h	2.46 × 10^6^	2.13
10.0 g/L scleroglucan treated for 6 h	2.42 × 10^6^	2.84
1.0 g/L scleroglucan treated for 8 h	3.26 × 10^6^	0.06
2.5 g/L scleroglucan treated for 8 h	3.64 × 10^6^	0.49
5.0 g/L scleroglucan treated for 8 h	1.60 × 10^6^	3.72
7.5 g/L scleroglucan treated for 8 h	2.31 × 10^6^	2.12
10.0 g/L scleroglucan treated for 8 h	2.07 × 10^6^	2.84
1.0 g/L scleroglucan treated for 10 h	/^a^	/^a^
2.5 g/L scleroglucan treated for 10 h	4.61 × 10^5^	4.80
5.0 g/L scleroglucan treated for 10 h	8.88 × 10^5^	2.55
7.5 g/L scleroglucan treated for 10 h	2.53 × 10^6^	1.50
10.0 g/L scleroglucan treated for 10 h	2.29 × 10^6^	3.05

a The molecular weight of the 1.0 g/L scleroglucan sample was 321 Da and its peak proportion was 71.23% after treatment with the HCl-HPH method (the time of HCl pretreatment was 10 h).

## Conclusion

In this study, the decrease in scleroglucan production during the fermentation process was resolved by adjusting the state of the seeds inoculated. Additionally, by establishing a two-dose fed-batch mode, the production of scleroglucan was further enhanced, reaching 66.6 g/L, with a 53.3% glucose conversion ratio and productivity of 0.40 g/(L·h). Furthermore, based on optimizing the conditions of HPH treatment and HCl treatment, a combination HCl-HPH method was established for modifying the molecular weight of scleroglucan, which produced scleroglucan of 4.61 × 10^5^ Da. These established methods could provide a reference for the biosynthesis and molecular weight modification of scleroglucan and other polysaccharides.

## Data Availability

The original contributions presented in the study are included in the article/Supplementary Material, further inquiries can be directed to the corresponding author.
