# Supplementary material for: Efficient Production of Scleroglucan by Sclerotium rolfsii and Insights Into Molecular Weight Modification by High-Pressure Homogenization
Source: Front Bioeng Biotechnol. 2021 Sep 3;9:748213. doi: 10.3389/fbioe.2021.748213 (PMC8448344; doi:10.3389/fbioe.2021.748213)
Supplement: Supplementary file 1 [file Table1.DOCX]

# Efficient production of scleroglucan by *Sclerotium rolfsii* and insights into molecular weight modification by high-pressure homogenization

Weizhu Zeng^a,b,c^, Junyi Wang^a,b,c,d#^, Xiaoyu Shan^a,b,c,d^, Shiqin Yu^a,c,d^, Jingwen Zhou^a,b,c,d*^

^a^ National Engineering Laboratory for Cereal Fermentation Technology, Jiangnan University, 1800 Lihu Road, Wuxi, Jiangsu 214122, China;

^b^ Science Center for Future Foods, Jiangnan University, 1800 Lihu Road, Wuxi, Jiangsu 214122, China;

^c^ Key Laboratory of Industrial Biotechnology, Ministry of Education, School of Biotechnology, Jiangnan University, 1800 Lihu Road, Wuxi, Jiangsu 214122, China;

^d^ Jiangsu Provisional Research Center for Bioactive Product Processing Technology, Jiangnan University, 1800 Lihu Road, Wuxi, Jiangsu 214122, China.

^*^ Corresponding authors.

Jingwen Zhou

National Engineering Laboratory for Cereal Fermentation Technology, Jiangnan University, 1800 Lihu Road, Wuxi, Jiangsu 214122, China

Phone: +86-510-85914317, Fax: +86-510-85914371

E-mail: zhoujw1982@jiangnan.edu.cn

^#^ This author contributed equally to this paper.

## Table S1 Molecular weight of scleroglucan and its peak proportion treated with different HCl concentrations only.

| HCl concentration | Molecular weight (Da) | Peak proportion (%) |
| --- | --- | --- |
| 0.00 mol/L | 3.20×10^8^ | 0.69 |
| 0.05 mol/L | 1.58×10^8^ | 11.00 |
| 0.10 mol/L | 1.28×10^8^ | 3.42 |
| 0.20 mol/L | 245 | 98.00 |
| 0.30 mol/L | 178 | 99.85 |
| 0.50 mol/L | 159 | 99.96 |

* The method of HCl treatment only was conducted at 60 ^o^C for 2 h.

## Table S2 Molecular weight of scleroglucan (10^5^-10^6^) and its peak proportion pretreated by different concentrations of HCl.

| HCl concentration | Molecular weight (Da) | Peak proportion (%) |
| --- | --- | --- |
| 0.05 mol/L | 5.87×10^6^ | 12.44 |
| 0.10 mol/L | 3.11×10^6^ | 2.25 |
| 0.20 mol/L | 1.62×10^6^ | 0.13 |
| 0.30 mol/L | 2.85×10^6^ | 0.15 |
| 0.50 mol/L | 5.31×10^6^ | 0.21 |

* The results were obtained based on the HCl-HPH combination method.

## Table S3 Molecular weight of scleroglucan (10^5^-10^6^) and its peak proportion pretreated with different concentrations of HCl at different temperatures.

| Conditions | Molecular weight (Da) | Peak proportion (%) |
| --- | --- | --- |
| 0.10 mol/L HCl at 60 ^o^C | 3.11×10^6^ | 2.25 |
| 0.10 mol/L HCl at 70 ^o^C | 2.92×10^6^ | 0.07 |
| 0.10 mol/L HCl at 80 ^o^C | 5.20×10^6^ | 0.22 |
| 0.10 mol/L HCl at 90 ^o^C | 3.13×10^6^ | 13.35 |
| 0.20 mol/L HCl at 60 ^o^C | 1.62×10^6^ | 0.13 |
| 0.20 mol/L HCl at 70 ^o^C | 2.79×10^6^ | 0.12 |
| 0.20 mol/L HCl at 80 ^o^C | 2.57×10^6^ | 0.59 |
| 0.20 mol/L HCl at 90 ^o^C | 1.32×10^6^ | 6.13 |
| 0.30 mol/L HCl at 60 ^o^C | 2.85×10^6^ | 0.15 |
| 0.30 mol/L HCl at 70 ^o^C | 2.79×10^6^ | 0.38 |
| 0.30 mol/L HCl at 80 ^o^C | 5.06×10^6^ | 0.60 |
| 0.30 mol/L HCl at 90 ^o^C | 4.16×10^6^ | 0.41 |

* The results were obtained by the HCl-HPH combination method based on the pretreatment with different HCl concentrations.
